# Date Vinegar: First Isolation of *Acetobacter* and Formulation of a Starter Culture

**DOI:** 10.3390/foods13091389

**Published:** 2024-04-30

**Authors:** Zahra S. Al-Kharousi, Zainab Al-Ramadhani, Fatma A. Al-Malki, Nasser Al-Habsi

**Affiliations:** Department of Food Science and Nutrition, College of Agricultural and Marine Sciences, Sultan Qaboos University, P.O. Box 34, Al-Khod 123, Oman; zainab1996m7md@gmail.com (Z.A.-R.); fatma-almalki@hotmail.com (F.A.A.-M.); habsin@squ.edu.om (N.A.-H.)

**Keywords:** acetic acid, *Acetobacter*, date vinegar, ethanol, fermentation, starter culture

## Abstract

There is a lack of scientific analysis and control over the production of date vinegar in Oman, despite its growing demand in the worldwide market. Traditional production of date vinegar may lead to elevated amounts of ethanol (≥0.5%) and reduced content of acetic acid (<4%) compared to the standard acceptable levels. This study aimed to isolate non-*Gluconobacter* species from date vinegar produced by spontaneous fermentation and formulate starter cultures for quick and efficient production of date vinegar. In spontaneous fermentation date vinegar samples, the highest concentration of acetic acid was 10.42% on day 50. *Acetobacter malorum* (5 isolates), *A. persici* (3 isolates), and *A. tropicalis* (3 isolates) were identified based on 16S rRNA gene sequences for the first time in date vinegar. For date vinegar prepared with a starter culture of *Acetobacter* and yeast, the highest concentration of acetic acid was 4.67%. In conclusion, spontaneous fermentation resulted in the production of date vinegar with a high concentration of acetic acid, acceptable concentrations of ethanol and methanol, and the first isolation of three *Acetobacter* species. The formulated starter culture produced acceptable amounts of acetic acid and the time of fermentation was reduced 10 times (from 40 days to 4 days). This can provide the basis for producing a personalized or commercial product that ensures the production of good-quality date vinegar in an easier, faster, safer, and more efficient way from low-quality and surplus dates.

## 1. Introduction

Vinegar is a worldwide product and its usage dates back to more than 2000 years ago where it has been used to preserve and flavor foods, combat infections, heal wounds, decontaminate surfaces, manipulate diabetes [1], remove grease, and neutralize odors [2,3]. Vinegar can be produced from nearly any fermentable carbohydrate-raw material, such as apples, berries, dates, grapes, melon, and wine [4]. Low-quality dates characterized by being dark in color or black, small, and bruised with undesirable flavor [5] are unmarketable; however, their high content of sugars enables exploitation as a raw material for producing many value-added products, including liquid sugar, date syrup, date paste, and vinegar [6]. Date palm (*Phoenix dactylifera* L.) is the most important crop in Oman, in which it occupies 82% of all grown fruits in the country [7]. It is one of the earliest cultivated plants in the world that has been cultivated for five thousand years [8]. Date fruit consists of 70% carbohydrates, most of which are in the form of reduced sugars, mainly fructose and glucose, fat (1%), and protein (2%). Copper, magnesium, potassium, and selenium are present as major minerals in dates. Date fruit is also rich in vitamins B-complex and C and is classified as a high source of dietary fibers (8.0 g/100 g), and a good source of antioxidants, such as carotenoids and phenolics [9].

Vinegar can be produced from dates as its sugars are converted first to ethanol and then to acetic acid [4], which is the chemical that makes the product vinegar. Acetic acid gives vinegar a tart flavor and a pungent, biting odor. Diluted acetic acid should not be considered vinegar as stated by The US Food and Drug Administration (FDA). Vinegar also contains amino acids, mineral salts, vitamins, polyphenolic compounds, and non-volatile organic acids [1].

There are two steps in vinegar production. The first one is known as alcoholic fermentation, in which the yeasts convert sugars to alcohol in an anaerobic environment. The second one is acetous fermentation in which acetic acid bacteria (AAB) convert the produced alcohol into acetic acid in the presence of oxygen. Vinegar can be obtained by fast or slow fermentation processes. Rapid fermentation is attained by oxygenating the liquid and by submerging the bacterial culture. Slow vinegar production is used to produce traditional vinegar in which the AAB grow on the surface and the fermentation process lasts for weeks or months. This longer fermentation time permits the aggregation of yeasts and AAB into a nontoxic slime layer known as the mother of vinegar. Vinegar produced commercially is mostly filtered and pasteurized to prevent the formation of vinegar eels (nematode *Turbatrix aceti*) that feed on vinegar organisms and can be found in naturally produced vinegar [1].

AAB are Gram-negative or Gram variable, non-spore-forming ellipsoid to rod cells that can exist in single, pairs, or short chains. They may have peritrichous or polar flagella. They are catalase-positive and oxidase-negative. They are obligate aerobes in which oxygen is used as the terminal electron acceptor [10,11]. However, compounds other than oxygen can be used as final electron acceptors allowing AAB to survive in nearly anaerobic environments, such as during wine fermentation, even though they mainly can be present in a viable but non-culturable (VBNC) state [12]. AAB grow optimally at 28–30 °C, though some species are thermotolerant and their optimum pH is 5.0–6.5. Phenotypic, chemotaxonomic, and genotypic characterization have been used to identify the phylogenetic relationships among AAB and to describe new genera and species. However, sometimes it is difficult to differentiate AAB at the species level due to similarities in phenotypic characteristics as well as the high sequence similarity of the 16S rDNA of phylogenetically closely related species. The AAB belong to the order Rhodospirillales and the family Acetobacteraceae that involves 19 genera and 92 species according to a recent classification. The genera are *Acetobacter*, *Acidomonas*, *Ameyamaea*, *Asaia*, *Bombella*, *Commensalibacter*, *Endobacter*, *Gluconacetobacter*, *Gluconobacter*, *Granulibacter*, *Komagataeibacter*, *Kozakia*, *Nguyenibacter*, *Neoasaia*, *Neokomagataea*, *Saccharibacter*, *Swaminathania, Swingsia*, and *Tanticharoenia* [13,14]. Acetic acid bacteria are not easy to isolate and cultivate, especially from fermented beverages. Underestimation of species richness and low recovery could be due to the proportion of the population that can enter the VBNC state. In this case, genotypic methods can reveal higher bacterial diversity compared to the culturing techniques [11].

Yeasts are unicellular fungi with vegetative states that reproduce asexually by fission or budding and sexually without fruiting bodies. They inhabit various environments such as fruits and flowers, plant surfaces and exudates, insects and other invertebrates, birds, mammals, and highly diverse soils [15,16]. Several genera and species of yeasts play significant roles in the production of vinegar. Fermenting yeasts are responsible for the production of the alcoholic substrate from consuming carbohydrates. The main groups are yeasts belonging to the genus *Saccharomyces*, apiculate yeasts of the genera *Hanseniaspora* and *Kloeckera*, lactose-fermenting yeasts of the genus *Kluyveromyces*, and osmophilic yeasts of the genus *Zygosaccharomyces* [16].

Although numerous microbiological studies have been conducted to examine the process of vinegar fermentation, knowledge about microbial diversity and the roles involved in fermentation is still fragmentary and not systematic enough [17]. In Oman, vinegar is traditionally produced from dates with no known specific control of the process, which raises questions about the hygiene condition of the final product [4], or if the product can be defined as a true vinegar. Vinegar should not contain less than 4% acetic acid. The residual ethanol content must be less than 0.5% [18,19]. The quality of the final vinegar product depends on many factors, especially on the type and diversity of the starter culture but also the quality of the raw material, the production method, and aging [12,17]. The variety of raw materials used in the production of vinegar is very great, ranging from byproducts and agricultural surpluses to high-quality substrates. The quality standard defines up to ten types of vinegar, which include wine vinegar, fruit, cider, alcoholic, cereal, malt, malt distillate, balsamic (with added grape must), and other balsamic kinds of vinegar, which encompass any other substrate of agricultural origin, such as honey or rice.

The innovation of starter cultures for the improvement of traditional vinegar production is an important biotechnological advancement for vinegar quality and safety [20,21,22,23]. Local people in Oman always raise questions, especially on social media, on whether the quality of the final product of traditional date vinegar meets the required standards of ethanol and acetic acid concentrations. In our previous study [24], 12 homemade date vinegar samples ready for consumption were collected from different local producers. About 67% of the samples had an ethanol concentration of more than the standard level of 0.5% and only one sample reached the standard acetic acid concentration of more than 4%. Also, the simulation of the traditional process while preparing 28 lab-made vinegar samples did not guarantee the production of vinegar with acceptable ethanol and acetic acid concentrations. Forty-six percent of samples had ethanol concentrations above the standard limit and none of them had the required level of acetic acid. Moreover, both homemade and lab-made vinegar samples contained levels of methanol greater than the acceptable levels. Formulating a novel starter culture can help produce date vinegar containing acceptable amounts of ethanol, acetic acid, and methanol and thus improve its quality and safety.

In our previous research [24], only AAB belonging to the genus *Gluconobacter* were isolated and characterized. The main objective of this research was to isolate new species of AAB, especially other than *Gluconobacter,* from date vinegar produced through spontaneous fermentation by modifying the procedures as described in the methods, and formulate starter cultures that can be used to produce a good-quality date vinegar in a time shorter than 40 days which is mostly used for traditional spontaneous fermentation. This research may provide the local industry with the required information to develop and commercialize a good-quality date vinegar based on scientific background utilizing low-quality or surplus dates in vinegar production.

## 2. Materials and Methods

### 2.1. Preparation of Date Vinegar Samples through Spontaneous Fermentation

For the isolation of AAB, six date vinegar samples were prepared in the laboratory. A low-quality date (*Phoenix dactylifera* L.) variety ‘Um al Sila’ was used for this purpose and it was collected from a local farm in Al-Suwaiq, Al-Batinah Governorate, Oman. The samples were mixed with sterile distilled water in a ratio of 1:4 (*w*/*v*), 175 g date fruit and 525 mL distilled water, in a stomacher bag and homogenized using a stomacher (Bagmixer 100 MiniMix, Interscience, Bois Arpents, France) for 1 min. After that, date broths were sieved into conical flasks and closed with sterile cotton plugs. All samples were incubated at 30 °C in an incubator (Gallen Kamp, Cambridge, UK) statically to permit spontaneous fermentation.

### 2.2. Microbiological Analysis

Samples were processed aseptically in a safety cabinet (Purifier class II, Labconco, Kansas, MO, USA). The microbial analyses were performed at 0, 10, 20, 30, and 40 days. All media and chemicals were from Oxoid, the UK, except if specified.

#### 2.2.1. Isolation of AAB and Yeasts

Two types of media, acetic acid bacteria-selective agar (ABS) and reinforced acetic acid and ethanol (RAE), were tested to check if they could support better growth of different isolates of AAB that were previously isolated from date vinegar on glucose yeast extract peptone ethanol calcium carbonate (GYPEC) medium, as all isolates (5 species) belonged to only one genus, *Gluconobacter* [24]. Moreover, the growth of AAB from unpasteurized commercial apple cider vinegar with the mother culture was tested. ABS medium contained 50 g D-(+)Glucose (Sigma Aldrich, Munich, Germany), 10 g yeast extract, 20 mg bromophenol blue (Fluka, Buchs, Switzerland), 20 g bacteriological agar, 1 mL glacial acetic acid (Sigma Aldrich, Germany), 50 mL absolute ethanol (Sigma Aldrich, Germany), 6 mg oxacillin (oxacillin sodium salt, Sigma-Aldrich, Beijing, China), and 1 L distilled water [25]. RAE medium contained 40 g glucose, 10 g peptone, 10 g yeast extract, 1.5 g citric acid (AnalaR, Louis, MO, USA), 3.38 g Na_2_HPO_4_ × 2 H_2_O (AnalaR, Louis, MO, USA), 10 g glacial acetic acid, 10 g absolute ethanol, and 980 g distilled water [26]. GYPEC included 20 g D-glucose, 8 g yeast extract, 5 g peptone, 3 g CaCO_3_ (GPR, VWR, Leuven, Belgium), 15 g bacteriological agar, 0.022 g bromocresol green (Sigma-Aldrich, St. Louis, MO, USA), 5 mL absolute ethanol, and 1 L distilled water. Different *Gluconobacter* isolates previously obtained from date vinegar were streaked on GYPEC, ABS, and RAE and incubated aerobically at 30 °C for 2–3 days. The optimum bacterial growth (faster with bigger colonies) occurred on the GYPEC medium. Therefore, this medium was used for subsequent experiments. 

In addition, an enrichment broth was used for the isolation of AAB before subculturing on the solid GYPEC medium. The enrichment medium contained 1% D-glucose (*w*/*v*), 0.5% ethanol (*v*/*v*), 1.5% peptone (*w*/*v*), 0.8% yeast extract (*w*/*v*), 0.3% acetic acid (*v*/*v*), and 0.01% cycloheximide (*w*/*v*, Sigma-Aldrich, Beijing, China). Unlike our previous study in which the pH was adjusted to 3.5, the pH of the enrichment broth in this study was not adjusted and kept at 4.8. In addition, sterile filtered oxacillin (6 µg/mL, oxacillin sodium salt, Sigma-Aldrich, China) was added to the enrichment broth to inhibit the growth of lactic acid bacteria. Then, 1 mL of the sample was mixed with 5 mL of the enrichment medium and incubated at 30 °C for 3 days. This was done in triplicate. After incubation, a loopful of each enrichment tube was streaked on GYPEC medium and incubated at 30 °C for 2–5 days. Bacteria that produced clear haloes on GYPEC medium and changed the green color of bromocresol green to yellow, as an indicator of the production of acetic acid, or reversed the yellow color to green due to the oxidation of acetic acid after 2–3 days of incubation were presumptively identified as AAB. Some chemical tests were used for the presumptive confirmation of AAB. A catalase test was performed by mixing a drop of 3% H_2_O_2_ with some bacterial colonies on a glass slide and observing the formation of O_2_ bubbles, which indicates hydrolysis of H_2_O_2_ by bacterial catalase. The Gram-reaction test was performed by mixing a drop of 3% KOH with some bacterial colonies on a glass slide. Formation of a DNA thread when the loop used for mixing was raised was considered as indicative that the bacteria were Gram-negative, while if the mixture remained watery, the bacteria were considered Gram-positive. The oxidase test was conducted following the manufacturer’s instructions (Oxoid, UK) by touching bacterial colonies on strips containing the reagent tetramethyl-p-phenylenediamine and checking the formation of the dark purple color of indophenol if the bacteria possess oxidase. Gram stain was conducted and then the morphological characteristics of AAB cells were observed under the light microscope to confirm that they were Gram-negative and rod-shaped [24]. According to their morphological characteristics, eleven isolates were selected and purified on GYPEC medium not containing bromocresol green. The pure colonies were preserved in cryogenic vials with beads (Viabank, Corsham, Wiltshire, UK) at −80 °C for identification. All of them showed the phenotypic characteristic of the appearance of clear haloes around bacterial colonies, changing the color of media from green to yellow and returning to green after about 2 days. Some isolates showed the morphological growth characteristics of *Gluconobacter*, characterized by retaining the yellow color during the incubation period (up to 7 days) and not returning to the green color. These isolates were not processed further as similar ones have been previously studied, and this investigation focused on AAB other than *Gluconobacter.*

The presence of yeasts was checked by culturing samples on potato dextrose agar (PDA) using the spread plate method [27]. To suppress the growth of bacteria, the medium was acidified to pH 3.5 by adding 1 mL of 10% lactic acid (ThermoFisher Scientific, Waltham, MA, USA) to every 100 mL of the sterilized medium at 50 °C. Triplicate plates were prepared from each dilution and then incubated at 25 °C for 3–4 days. The yeasts were isolated and purified on PDA and preserved in cryogenic vials with beads (Viabank, UK) at −80 °C. Yeasts in date vinegar have been previously studied and the results were reported [24]. 

##### Identification of Acetic Acid Bacteria

DNA was extracted using foodproof^®^StarPrep Two Kit (Biotecon Diagnostics GmbH, Potsdam, Germany) according to the manufacturer’s guidelines. The pure colonies were subcultured on GYPEC plates and then some colonies were transferred to a 1.5 mL reaction tube containing 300 µL lysis buffer. The contents of the tubes were mixed using a vortex (Stuart, UK). Sterilized glass beads were used to disrupt the bacterial cells in the reaction tubes that were then incubated in a water bath (Sub Aqua Plus, Cambridge, UK) at 95–100 °C for 5 min. After cooling, the reaction suspensions were mixed and centrifuged (Minispin, Eppendorf, Hamburg, Germany) at 13,000× *g* for 5 min. The DNA was collected from the supernatant. The quality and quantity of the DNA were checked using NanoDrop^TM^ 2000 (Thermo scientific, Waltham, MA, USA).

Polymerase chain reaction (PCR) targeted the 16S rRNA gene of AAB and was performed as previously described [18]. Briefly, the PCR was done by transferring 1 µL of each primer (27F (forward) and 1492R (reverse); DNA sequences: 5′-AGAGTTTGATCMTGGCTCAG-3′ and 5′-TACGGYTACCTTGTTACGACTT-3′, respectively), 22 µL of Milli-Q water, and 1 µL of the DNA of AAB to PCR reaction tubes containing PCR beads (puReTaq Ready-To-Go PCR beads, GE Healthcare, Nightingales Lane, UK). The final volume was 25 µL. The negative control mixture for the PCR contained Milli-Q water instead of DNA. The thermal profile (Veriti 96-well Thermal cycler, Applied Biosystems, Singapore) for the PCR was as follows: stage 1, denaturation at 95 °C for 2 min; stage 2, denaturation at 95 °C for 30 s, annealing at 54 °C for 30 s, extension at 72 °C for 1 min (35 cycles); and stage 3, final extension at 72 °C for 10 min and then kept at 4 °C. 

A total of 1.5% agarose gel was prepared by mixing 1.5 g agarose (Thermo Scientific, TopVision, Waltham, MA, USA) with 100 mL of 0.5× TBE buffer (Tris/Borate/EDTA) and dissolving in the microwave. After cooling, 3 µL of 0.5 µg/mL ethidium bromide (Sigma-Aldrich, St. Louis, MO, USA) was added. The gel was poured into a casting tray. After solidification, the solid gel was placed into a chamber filled with 0.5× TBE buffer. Then, 5 µL aliquots of the PCR products were mixed with 2 µL of DNA loading dye (6× DNA Loading Dye, ThermoFisher Scientific) to visualize the movement of the DNA through the gel, and then pipetted into the row wells at the top of the gel slab. The negative and positive leads were connected to the chamber and to a power supply where the voltage was set (voltage: 120 V, current: 400 A, time: 35 min). A 100 bp ladder (Fermetas, O’RangeRuler, ThermoFisher Scientific) was run on each gel as a reference for sizes. Gels were visualized by UV using GelDoc (GeneFlash, Syngene, Cambridge, MA, USA). 

Appropriate PCR products were sequenced (Macrogen, Seoul, Republic of Korea) using the same primers used for amplification. DNA sequences were analyzed through ‘ChromasPro’ program (version 2.1.10.1, 2003–2021, Technelysium Pty Ltd., South Brisbane, Queensland, Australia). Sequences of the products of both primers were assembled into a contiguous consensus. The sequences as presented in the chromatogram were edited using the sequence editor. The low-quality sequences in the right and left trim locations were cleared. The sequences were aligned and compared online with those found in the ‘National Centre for Biotechnology Information’ (NCBI) using the ‘Basic Local Alignment Search Tool’ (BLAST) program (http://www.ncbi.nlm.nih.gov/; accessed on 15 January 2024). The sequences of AAB were submitted to GenBank to be assigned accession numbers.

##### Phylogenetic Analysis

Phylogenetic analysis of AAB was performed based on the 16S rRNA gene sequences. MUSCLE was used for the alignment of sequences. MEGA11 [28] was used to construct the phylogenetic trees using the neighbor-joining method. The evolutionary distances were computed using the Kimura 2-parameter method (1000 replicates) after testing the best method in Mega 11 by checking the values of the Bayesian information criterion (BIC) and corrected Akaike information criterion (AICc) as previously described [28,29]. 

### 2.3. Chemical Analysis 

Chemical analysis was performed at 0, 10, 20, 30, and 40 days of fermentation. The samples were analyzed for total soluble solids (°brix), pH, glucose, fructose, methanol, ethanol, and acetic acid. In addition, analyses of ethanol, methanol, and acetic acid were conducted on day 50. 

The pH of the samples was measured using a pH meter (Metrohm, 744 pH meter) after calibration. Determination of total soluble solids (°brix) was done using an electronic refractometer (ATAGO, Tokyo, Japan). The contents of acetic acid (CH_3_COOH), methanol (CH_3_OH), and ethanol (C_2_H_6_O) were determined using headspace (HS-20 Loop Model), gas chromatography (AGILENT-7890A, Santa Clara, CA, USA) with flame ionization detection (HS-GC-FID), as has been described previously [24]. In brief, date vinegar samples were filtered using filter paper (185 mm, Whatman). For sample introduction, the Agilent 7890A-GC-Agilent-7697A HSS Loop headspace sampler was used in the static-loop headspace mode. The injection volume was 1 µL, the inlet temperature was 100 °C, and the split ratio was 5:1. Effluent from the HS-20 was split 20-to-1 and then divided into two identical columns using a 3-way “T” fitting. The type of the column was Supelco-23473.0—325 °C, 30 m × 250 µm × 0.5 µm. The mobile phase flow rate (He) was 1 mL/min. The oven temperature was 60 °C, and then it was held for 1 min. The temperature ramp was 10 °C per min until 150 °C, then it was held at 150 °C for 10 min, and the run time was 150 min. The outlet ends of the two columns were connected to the FID detectors. The detector temperature was 250 °C and the detector hydrogen flow was 30 mL/min. The zero air was 400 mL/min. Analysis was done using the Software “Chemstation”. Glucose and fructose analysis was performed using high-performance liquid chromatography (HPLC), following a previous method [30]. In short, an HPLC (Nexera UHPLC/HPLC, Tokyo, Japan) equipped with a Unison UK-Amino column (150 × 3 mm) with a refractive index detector was utilized. Standards of glucose and fructose were prepared with a stock concentration of 0.1g/mL and then diluted five times (100 ppm, 200 ppm, 300 ppm, 400 ppm, and 500 ppm) and the sample concentration was 0.1 g/100 mL. Acetonitrile was used as a mobile phase with a ratio of (75:25) (acetonitrile:water) at 40 °C at a flow rate of 0.3 mL/min with a 10 µL injected sample. Standard curves were drawn for glucose and fructose, and the concentrations of sugars were determined.

### 2.4. Starter Culture Samples

#### 2.4.1. Preparation of Starter Cultures

Three samples were prepared for inoculation with starter cultures. Three different starter cultures were prepared as described in Table 1. Each bacterium was inoculated from the preserved bead into 5 mL glucose yeast peptone (GYP) medium (10% glucose, 5% yeast extract, 3% peptone prepared in distilled water) and incubated for 48 h at 30 °C. Yeast isolate was subcultured onto yeast extract peptone (YP) medium (1% yeast extract, 2% peptone, and 2% glucose prepared in distilled water) and incubated for 48–72 h at 25 °C. The glucose solution was sterilized by filtration (syringe filter, 0.25 µm) and added to the broth after sterilization. The turbidity was checked for each type of broth [31]. After 48 h incubation, starter culture 1 was prepared by mixing 1 mL of each selected bacterial growth with 1 mL of yeast growth while starter cultures 2 and 3 were prepared by mixing 2 mL of each selected bacterial growth with 1 mL of yeast growth (Table 1).

#### 2.4.2. Inoculation of Date Broth with Formulated Starter Cultures

Three samples were prepared with formulated starter cultures as shown in Table 1. The samples of date broth were prepared by mixing dates with distilled water in a ratio of 1:4 (*w*/*v*): 175 g of date and 525 mL of sterilized distilled water. The mixture was homogenized in a stomacher bag, double-filtered using a sieve, and transferred into flasks. The samples were inoculated with the starter culture (7 mL) as described in Table 1. All samples were incubated at 30 °C and care was taken to avoid shaking samples to allow AAB to ferment statically.

#### 2.4.3. Sample Analyses

The samples were analyzed chemically by measuring °brix, pH, glucose, fructose, ethanol, methanol, and acetic acid at 0, 1, and 4 days as described for spontaneous samples in Section 2.3. Microbial analyses were conducted by observing the growth of AAB and yeasts in all samples in the mentioned days of fermentation, and culturing AAB on the GYPEC medium and yeast on the PDA medium to check their viability.

### 2.5. Data Analysis

Statistical analysis of the data was done using the SAS statistical software package (JMP^®^ SAS 17.2.0, 2022–2023, Cary, NC, USA). One-way analysis of variance (ANOVA) was used to study if there were significant differences between different parameters (pH, °brix, glucose, fructose, methanol, ethanol, and acetic acid) according to the method of fermentation (spontaneous or starter culture inoculation). Differences were considered significant if *p* < 0.05. Moreover, the data were analyzed using a multivariate analysis approach to perform principal component analysis (PCA) [32] to study correlation patterns between different parameters.

## 3. Results

### 3.1. Spontaneous Fermentation

#### 3.1.1. Chemical Parameters

**Figure 1:**
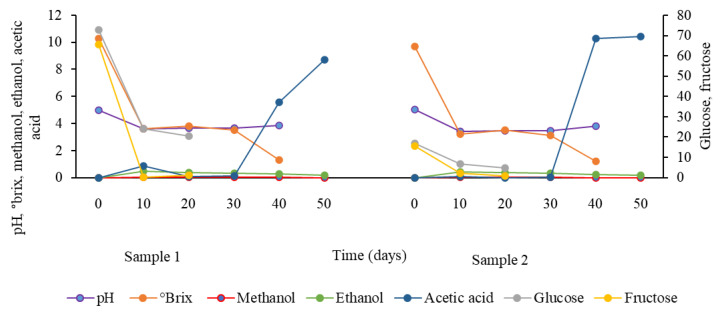
Changes in pH, °brix, glucose (mg/g), fructose (mg/g), ethanol (%), methanol (%), and acetic acid (%) throughout the spontaneous fermentations in date vinegar samples 1 and 2.

The results of parameters linked to spontaneous fermentation are represented in Figure 1 for samples 1 and 2. The other four samples were not analyzed further as it was not possible to isolate AAB from them. The pH declined with time and the lowest value was 3.41 in sample 2 after 10 days. Total soluble solid contents (°brix) decreased with time until they reached 1.3 and 1.2 in samples 1 and 2, respectively, after 40 days of fermentation. The reduction in glucose and fructose concentrations was observed in both samples during the experiment. Methanol and ethanol contents rose until around 30–40 days, then they dropped down to 0.0038 and 0.0018 for methanol and 0.193 and 0.186% for ethanol on day 50 for samples 1 and 2, respectively. There was a dramatic increase in the concentration of acetic acid on days 40 and 50 for both samples reaching 5.57 and 10.26 on day 40 and 8.7 and 10.42 in samples 1 and 2, respectively.

#### 3.1.2. Identification of Acetic Acid Bacteria and Genetic Analysis

**Figure 2:**
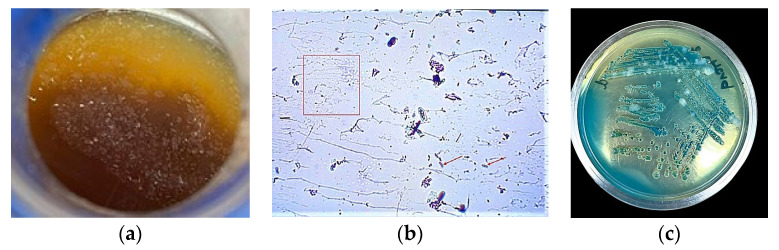
The mother of vinegar containing AAB and extracellular materials formed on the top layer of sample 2 during spontaneous fermentation (**a**). A microscopic picture of a Gram-stained film (mother of vinegar) shows AAB stained red (arrows) with the appearance of filaments (e.g., inside rectangle) of extracellular matrix produced by AAB (**b**). Mixed growth of various *Acetobacter* and *Gluconobacter* on GYPEC subcultured from the enrichment broth showing colonies with various shades of blue/green color (**c**).

As the fermentation process proceeded, a film formed at the top layer of the fermentation samples (Figure 2a). Gram staining of a portion of this film is shown in Figure 2b in which Gram-negative bacteria and filaments as components of the mother culture could be seen. Subculturing from the enrichment broth onto GYPEC gave colonies with various shades of the blue–green color (Figure 2c). From these, eleven AAB showed the phenotypic characteristic of the appearance of clear haloes around bacterial colonies, changing the color of media from green to yellow, and returning to green after about 2 days. These isolates were identified genotypically. The isolates that showed the morphological growth characteristic of retaining the yellow color during the incubation period (up to 7 days) and not returning to the green color have been previously confirmed to be *Gluconobacter* and the results were reported [24].

Three species of *Acetobacter* were identified: *Acetobacter malorum* (5 isolates), *A. persici* (3 isolates), and *A. tropicalis* (3 isolates). The percentage identity and accession numbers of these isolates are presented in Table 2. To our knowledge, this is the first isolation of the *Acetobacter* genus from date vinegar.

**Figure 3:**
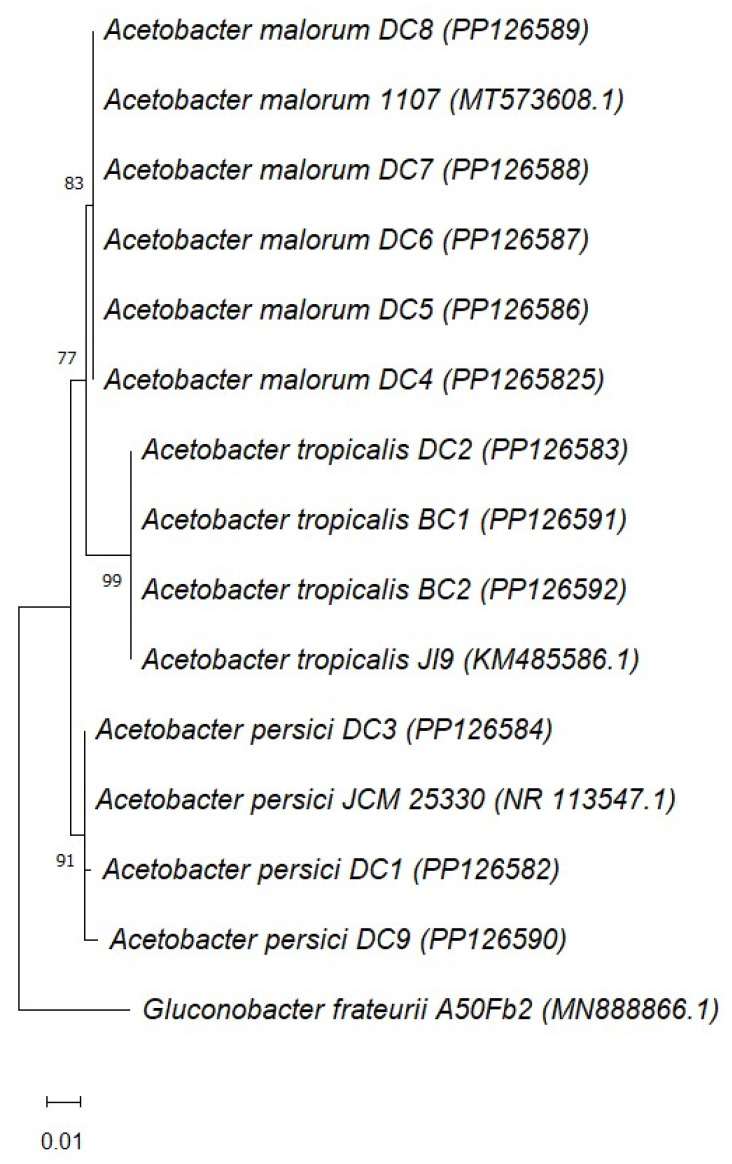
Neighbor-joining tree based on sequencing 16S rRNA gene for *Acetobacter* strains isolated from date vinegar. Accession numbers of the sequences of the isolates are shown in parentheses. Sequences of *A. malorum* 1107, *A. tropicalis* J19, and *A. persici* JCM 25330 have been retrieved from GenBank. *Gluconobacter frateurii* A50Fb2 (MN888866.1, isolated from date vinegar) was included as an outgroup. The Kimura 2-parameter method was used to compute evolutionary distances (1000 replicates in the bootstrap test).

The phylogenetic analysis of *Acetobacter* based on 16S rRNA gene sequences is shown in Figure 3. Each species, *A. malorum*, *A. tropicalis*, and *A. persici*, made a distinct clear cluster with strong bootstrap values (83, 99, and 91, respectively). The sequences of the three species that were retrieved from the GenBank clustered also with their respective species isolated in this study.

### 3.2. Starter Cultures Samples

**Figure 4:**
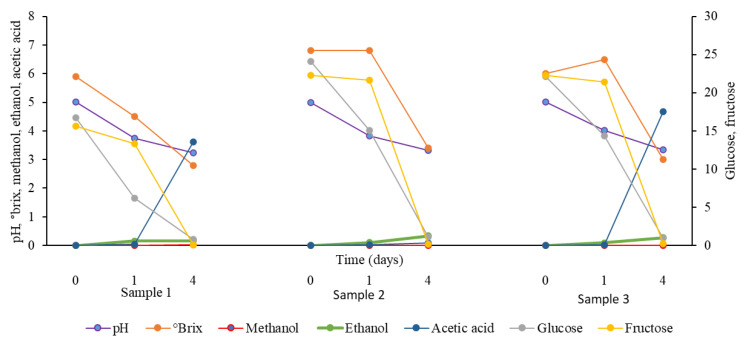
Changes in pH, °brix, glucose (mg/g), fructose (mg/g), ethanol (%), methanol (%), and acetic acid (%) throughout fermentation in samples 1, 2, and 3 inoculated with starter cultures 1 (*Acetobacter*, *Glucononbacter*, and yeast), 2 (*Gluconobacter* and yeast), and 3 (*Acetobacter* and yeast), respectively.

The growth of AAB and yeasts in all samples was confirmed by subculturing from samples on appropriate media on days 1 and 4 of the fermentation process. The results of different parameters are shown in Figure 4. The °brix and pH values decreased with time. The pH decreased to 3.23, 3.31, and 3.34 on day 4 for samples 1, 2, and 3, respectively. °Brix declined to 2.8, 3.4, and 3.0 in samples 1, 2, and 3, respectively. Likewise, the glucose and fructose concentrations decreased with time. Methanol concentration remained low and did not exceed 0.2% in all samples. There was a slight increase in the content of ethanol with time in all samples. A dramatic increase in the concentration of acetic acid occurred in samples 1 (inoculated with A5, A7, A32, BC1, DC3, DC4, and Y9) and 3 (inoculated with BC1, DC3, DC4, and Y9) that reached 3.62% and 4.67%, respectively, on day 4. However, the concentration of acetic acid was low in sample 2 which received the starter culture A5, A7, A32, and Y9, in which its concentration was 0.09% on day 4. The appearance of sample 3 with date vinegar produced using a formulated starter culture after 4 days of fermentation is shown in Figure S1.

**Figure 5:**
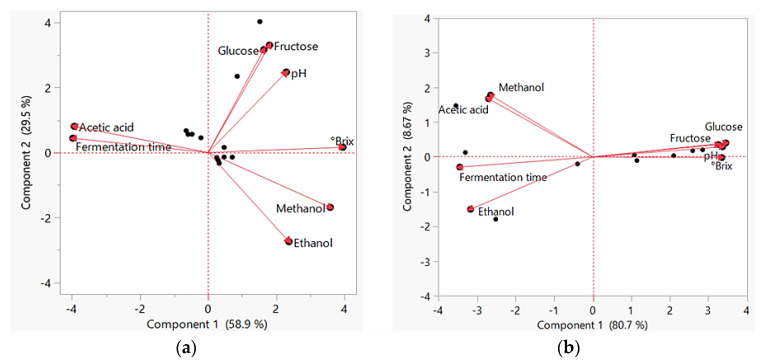
Principal component analysis (PCA) biplot performed with pH, °brix, glucose, fructose, ethanol, methanol, and acetic acid concentrations, and fermentation time for vinegar samples prepared through (**a**) spontaneous fermentation and (**b**) inoculation with starter cultures.

Statistically, ANOVA showed that there were no significant differences between pH, °brix, glucose, fructose, methanol, and acetic acid in samples prepared with spontaneous fermentation or using starter cultures (*p* = 0.6123, 0.5265, 0.1722, 0.8975, 0.4236, 0.2019, α = 0.05). However, a significant difference in ethanol concentrations was detected (*p* = 0.0361, α = 0.05). The PCAs for spontaneous fermentation samples and starter culture samples are shown in Figure 5. For the former, acetic acid concentrations positively correlated with the fermentation time (Pearson’s correlation: 0.9883, α = 0.05) which both negatively correlated with the methanol (Pearson’s correlation: −0.9497, −0.9028, respectively, α = 0.05) and ethanol concentrations (Pearson’s correlation: −0.6819, −0.6273, respectively, α = 0.05). The pH, °brix, glucose, and fructose concentrations correlated positively (Table S1). For samples with starter cultures, fermentation time positively correlated with the concentrations of acetic acid, methanol, and ethanol (Pearson’s correlation: 0.7358, 0.6573, 0.9100, respectively, α = 0.05) and negatively with pH, °brix, glucose, and fructose concentrations (Pearson’s correlation: −0.8873, −0.8990, −0.9164, −0.9370, respectively, α = 0.05). The pH, °brix, glucose, and fructose concentrations correlated positively (Table S2).

## 4. Discussion

### 4.1. Spontaneous Fermentation Samples

This study was designed to investigate the presence of AAB other than *Gluconobacter* that have been previously reported in date vinegar obtained by spontaneous fermentation, and to test the efficiency of these bacteria in producing date vinegar with a reasonable concentration of acetic acid. The lowest pH (3.41) presented in this study was higher than what was reported in date vinegar in other previous studies [24,33], which was 3.04 and 2.99%, respectively. The decrease in pH over the time of fermentation can be attributed to the accumulation of acetic acid or other acids secreted by AAB, lactic acid bacteria, and yeasts [23]. The decrease in the concentration of total soluble solids correlated with the decrease in the concentrations of glucose and fructose in both samples 1 and 2 with a starting °brix value of 10.3 and 9.7, respectively, as compared to 20 °brix which gave the highest ethanol production of 77.6 g/L in date vinegar studies previously [4]. This was also demonstrated by the positive correlation between pH, °brix, glucose, and fructose concentrations which negatively correlated with the fermentation time and acetic acid concentration, as the sugars were consumed during the fermentation and acetic acid was produced (Figure 5). Although glucose and fructose concentrations decreased with time in samples 1 and 2, the content of both sugars was higher in the raw material of sample 1 than in sample 2. However, the concentration of acetic acid was higher in sample 2 than in sample 1 on days 40 and 50. This may indicate that the decreased osmolarity of sample 2 created a better environment for acetic acid production by AAB.

The highest concentration of acetic acid attained in this study was very high in both samples 1 and 2 (8.7 and 10.42%, respectively) as compared to other studies which reported concentrations of 3.18% in some commercial date vinegar samples consumed in Iraq [33], 3.46% in samples prepared with spontaneous fermentation [18], and 6.0% in traditional date vinegar [34]. This might be related to the type of AAB present in the raw material—for example, a study [24] detected only *Gluconobacter* and no *Acetobacter* species in date vinegar. Thus, the amount of acetic acid in the current study was greater than the standard recommended level which should not be less than 4% [18,19], and this highlights the efficiency of the bacteria present in the raw material used in this study in producing high-quality date vinegar. After 10 days of fermentation, the amount of ethanol in this study in both samples 1 and 2 complied with the standard recommended level that should be less than 0.5% [18,19]. This is in contrast with previous studies that showed higher ethanol concentrations reaching 7.81% [24] and 2.53% [33].

Investigating the concentration of methanol is important, as methanol poisoning can occur due to fermentation errors that lead to the production of high levels of methanol [35,36]. The European Union (EU) general limit for naturally occurring methanol is 10 g methanol/L ethanol or 0.4% (*v*/*v*) methanol at 40% alcohol volume [37]. Although, studies are needed to determine the safe standard level of methanol in date vinegar; however, according to the acceptable range of methanol in wine vinegar (0.002–0.009%) and in cider vinegar (0.004–0.038%) [38], on day 40 of the fermentation, the concentrations of methanol in both samples 1 and 2 with spontaneous fermentation were acceptable (0.007 and 0.003%, respectively). However, sometimes throughout the fermentation process, the concentrations slightly exceeded these limits. Nevertheless, the highest percentage of methanol in this study (0.0239%) was less than the highest percentage of 0.35% reported previously in date vinegar [24]. Yeast, fungi, and bacteria that possess pectinesterase may cause partial hydrolysis of pectin to pectic acid and methanol, and certain strains of *S. cerevisiae* can produce methanol. Thus, the type of raw material that contains a lesser amount of pectin, or using starter cultures or mother of vinegar that contains microbial strains that do not produce methanol may help reduce the amount of methanol produced [36].

To our knowledge, this is the first investigation to reveal the presence of three species belonging to the genus *Acetobacter* in date vinegar obtained by spontaneous fermentation. The most abundant species was *A. malorum* (5 isolates) followed by *A. persici*, and *A. tropicalis* (3 isolates each). Bacteria belonging to each species of *A. malorum*, *A. persici*, and *A. tropicalis* clustered together with strong bootstrap values (83, 99, and 91, respectively) along with their respective species that were retrieved from the GenBank (Figure 3). *A. malorum* was reported in Korean traditional vinegar prepared from *Rubus coreanus* fruits [39]. The examination of various flowers, fruits, mushrooms, and fermented rice products gathered in Thailand revealed the presence of different strains of AAB including *A. persici*, and *A. tropicalis* [40]. AAB are considered fastidious as they have lower cultivability, and many strains lose some features when they are grown in culture media [12]. In one study [41], 64 strains of AAB were isolated from Indonesian sources such as fruits, flowers, and fermented foods after an enrichment step. In this research, the incorporation of cycloheximide and oxacillin was necessary to inhibit yeasts and lactic acid bacteria, respectively, and allow for the recovery of AAB. Large colonies of lactic acid bacteria were observed growing and obscuring AAB on GYPEC plates inoculated from enrichment broth not containing oxacillin (preliminary experiments).

### 4.2. Starter Culture Samples

The results showed that the addition of formulated starter cultures to date broth can help accelerate the fermentation process by the production of ethanol by yeast (Y9) in the alcoholic stage and the quick conversion of alcohol to acetic acid in the acetous stage with the help of AAB in less than one week. The small positive correlation between acetic acid and ethanol (Figure 5, Table S2) might indicate this quick conversion. However, the concentration of ethanol was acceptable (<0.5%) in all inoculated samples [18,19]. The pH, °brix, glucose, and fructose concentrations correlated to each other positively and negatively to ethanol, acetic acid, and the fermentation time as the sugars were consumed and ethanol and acetic acid were produced with time. Statistical similarities showed by ANOVA between various parameters in spontaneous vinegar samples and samples inoculated with starter cultures indicate the efficiency of the starter cultures in producing good-quality vinegar like the one produced by spontaneous fermentation and containing acceptable concentrations of acetic acid.

Differences in concentrations of acetic acid between samples 1, 2, and 3 can be attributed to the type of bacterial strains used for starter culture preparation. Sample 1 contained yeast, *Gluconobacter*, and *Acetobacter*, sample 2 contained yeast and *Gluconobacter*, while sample 3 contained yeast and *Acetobacter* (Table 1). The highest content of acetic acid found in sample 3 (4.67%), inoculated with yeast and *Acetobacter*, was greater than the standard recommended level, not less than 4% [18,19]. However, this was less than that which was previously reported [42] in date vinegar (6.62%) produced by inoculation with *S. cerevisiae* at the first stage and AAB from old vinegar at the second stage of the acidification process, and less than that attained in the current study by spontaneous fermentation after 40 and 50 days fermentation in samples 1 and 2 (highest concentrations 8.7 and 10.42%, respectively). *A. malorum* was previously reported to produce the highest concentration of acetic acid besides *A. pasterianus* after 5 days of fermentation at 30 °C, but in an artificial medium containing glucose, glycerol, polypeptone, yeast extract, potato extract, acetic acid, and ethanol [33]. Another study [43] attempted to select thermotolerant AAB with no overoxidation ability. The AAB were collected from palm wine and mango pulp after fermentation. Three strains identified as *Gluconobacter oxydans* (Ski1), and *Acetobacter ghanensis* (Fke 22 and Fk5) produced up to 10% acetic acid at 37 °C. However, the test medium was an artificial one containing ethanol, yeast extract, peptone, Na_2_HPO_4_, and MgSO_4_.

Phenotypically, the isolates of *Acetobacter* (BC1, DC3, DC4) that were used to produce the starter culture showed the pattern of overoxidation of acetic acid, which usually results in its conversion to carbon dioxide and water. Thus, following acetic acid production by these isolates in shorter periods (hours) may allow a better understanding of the fermentation dynamics, because the production of acetic acid may reach a peak and then drop. Some researchers [37] found that certain strains of *Acetobacter* consume acetic acid accumulated in the culture for vinegar fermentation when all available carbon and energy sources are exhausted in the medium and only acetic acid remains in the late stationary phase. These researchers observed AAB rapid growth showing a second stationary phase and a typical biphasic growth curve. It was also found that the cells from the first growth phase were acid tolerant, while the cells from the second growth phase became acid sensitive, and no acetate oxidation occurred in vinegar containing more than 4.5% acetic acid. In addition, there was a threshold for acetate concentration as their selected *Acetobacter* strains oxidized acetate when the final concentration of acetic acid accumulated was less than 3.7%. They concluded that *Acetobacter* rapidly grew on acetic acid after ethanol exhaustion because acetic acid was converted to acetyl-CoA by acetyl-CoA synthetase, and then acetate was put in the TCA and glyoxylate cycles [44]. Ethanol concentration can also play a role in acetic acid overoxidation as the entry into the tricarboxylic acid cycle might be inhibited by the presence of low concentrations of ethanol of about 0.5% in vinegar [12].

Samples 1 and 2 that received the starter culture containing *Acetobacter* and *Gluconobacter* (sample 1) or *Gluconobacter* alone (sample 2) showed less production of acetic acid, though that of sample 1 was close to sample 3. Thus, the presence of *Acetobacter* (samples 1 and 3) was necessary to increase the production of acetic acid as compared to *Gluconobacter* (sample 2). This may also explain why none of the 28 lab-prepared date vinegar samples in our previous study [24] contained the acetic acid recommended level (4%), as only *Gluconobacter* strains were isolated. It seems that spontaneous fermentation is a complex phenomenon determined by many factors. For example, the microbiota of the fermentation vinegar medium is very diverse, and though dominated by a large number and types of yeasts, AAB, and lactic acid bacteria, some other microflora might also be present [24]. More studies will be needed to determine the influence of these microflora and their interaction to optimize the conditions to produce high-quality date vinegar in industry and by local producers. On the other hand, it was shown that the concentrations of phenolic compounds in prickly pear vinegar samples inoculated with *A. malorum* were higher than in *G. oxydans* prickly pear vinegar samples, which highlights the importance of the starter culture strain for the quality of the final product [45].

## 5. Conclusions

Acetic acid bacteria belonging to three different species were isolated from date vinegar produced by spontaneous fermentation for the first time. They were identified as *A. malorum*, *A. persici*, and *A. tropicalis* based on sequencing the 16S rRNA gene. These samples contained high acetic acid concentrations, reaching 10.42%. The formulated starter culture accelerated the fermentation process from 40–50 days to less than one week, with the highest production of acetic acid of 4.7% obtained with a starter culture containing *A. malorum*, *A. persici*, *A. tropicalis*, and *S. cerevisiae*. If needed, the content of acetic acid may be further increased by modifying the process of production. More studies can validate using the formulated starter culture which can benefit the industrial sector and the local producers of date vinegar. This ongoing research endeavors to isolate additional variants of AAB to enhance the production of date vinegar, while simultaneously developing and validating novel starter cultures. Subsequent investigations ought to encompass an in-depth analysis of diverse flavor and aromatic constituents, including phenolics, aldehydes, and amino acids as well as testing vinegar production using the formulated starter cultures in industrial conditions with a reasonable number of replications to ensure the consistency of results in industrial applications and for local producers.

## Figures and Tables

**Table 1:** The content of formulated starter cultures for each sample.

Bacteria Code	Bacteria Name	GenBank Accession Numbers
A5	*Gluconobacter kanchanaburiensis*	MN888815
A7	*Gluconobacter oxydans*	MN888816
A32	*Gluconobacter frateurii*	MN888833
DC3	*Acetobacter persici*	PP126584
DC4	*Acetobacter malorum*	MN888816
BC1	*Acetobacter tropicalis*	PP126591
Y9	*Saccharomyces cerevisiae*	MN888781
Starter culture	Content	
1	A5, A7, A32, DC3, DC4, BC1, and Y9	
2	A5, A7, A32, and Y9	
3	DC3, DC4, BC1, and Y9	

**Table 2:** Names, % identity, and accession numbers of acetic acid bacteria type strains (identified by PCR) isolated from date vinegar samples produced by spontaneous fermentation.

Seq.	Bacteria ID	Name of Bacteria	% Identity	GenBank Accession #
1	DC1	*Acetobacter persici*	99.75	PP126582
2	DC2	*Acetobacter tropicalis*	99.84	PP126583
3	DC3	*Acetobacter persici*	99.77	PP126584
4	DC4	*Acetobacter malorum*	99.84	PP126585
5	DC5	*Acetobacter malorum*	99.92	PP126586
6	DC6	*Acetobacter malorum*	100.00	PP126587
7	DC7	*Acetobacter malorum*	99.85	PP126588
8	DC8	*Acetobacter malorum*	99.77	PP126589
9	DC9	*Acetobacter persici*	99.27	PP126590
10	BC1	*Acetobacter tropicalis*	99.92	PP126591
11	BC2	*Acetobacter tropicalis*	99.85	PP126592

## Data Availability

The original contributions presented in the study are included in the article/Supplementary Materials; further inquiries can be directed to the corresponding author.

## Supplementary Materials

The following supporting information can be downloaded at: https://www.mdpi.com/article/10.3390/foods13091389/s1, Figure S1: Sample 3 of date vinegar produced using a formulated starter culture after 4 days of fermentation; Table S1: Pearson’s correlation values for different parameters for spontaneous vinegar samples; Table S2: Pearson’s correlation values for different parameters for vinegar samples inoculated with starter cultures.
