# Supplementary material for: Date Vinegar: First Isolation of Acetobacter and Formulation of a Starter Culture
Source: Foods. 2024 Apr 30;13(9):1389. doi: 10.3390/foods13091389 (PMC11083709; doi:10.3390/foods13091389)
Supplement: Supplementary file 1 [file foods-13-01389-s001.zip › foods-2918116-supplementary.pdf]

Date vinegar: Isolation of *Acetobacter* and formulation of a starter culture

Zahra S. Al-Kharousi[1*], Nasser Al-Habsi[1], Zainab Al-Ramadhani[1], Fatma A. Al-Malki[1]

[1]Department of Food Science and Nutrition, College of Agricultural and Marine Sciences, Sultan Qaboos University, P.O. Box: 34, Al-Khoud, Postal Code: 123, Muscat, Oman

*** Correspondence:** Zahra S. Al-Kharousi, email: umohaned@squ.edu.om

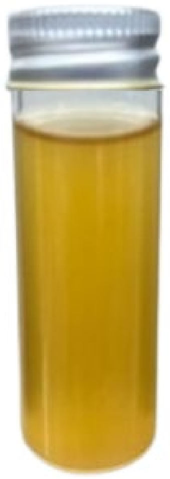

Figure S1. Sample 3 of date vinegar produced using a formulated starter culture after 4 days of fermentation.

Table S1. Pearson's correlation values for different parameters for spontaneous vinegar samples.

	Fermentation time	°Brix	pH	Glucose	Fructose	Methanol	Ethanol	Acetic acid
Fermentation time	1.0000	-0.9806	-0.5411	-0.2699	-0.3265	-0.9028	-0.6273	0.9883
°Brix	-0.9806	1.0000	0.6770	0.3472	0.4175	0.8466	0.4735	-0.9617
pH	-0.5411	0.6770	1.0000	0.4832	0.5728	0.2170	-0.2955	-0.4551
Glucose	-0.2699	0.3472	0.4832	1.0000	0.9561	0.0615	-0.1082	-0.2127
Fructose	-0.3265	0.4175	0.5728	0.9561	1.0000	0.0673	-0.1639	-0.2559
Methanol	-0.9028	0.8466	0.2170	0.0615	0.0673	1.0000	0.8108	-0.9497
Ethanol	-0.6273	0.4735	-0.2955	-0.1082	-0.1639	0.8108	1.0000	-0.6819
Acetic acid	0.9883	-0.9617	-0.4551	-0.2127	-0.2559	-0.9497	-0.6819	1.0000

Table S2. Pearson's correlation values for different parameters for vinegar samples inoculated with starter cultures.

	Fermentation time	°Brix	pH	Glucose	Fructose	Methanol	Ethanol	Acetic acid
Fermentation time	1.0000	-0.8990	-0.8873	-0.9164	-0.9370	0.6573	0.9100	0.7358
°Brix	-0.8990	1.0000	0.7571	0.9187	0.9715	-0.6497	-0.8063	-0.7380
pH	-0.8873	0.7571	1.0000	0.9254	0.7677	-0.7521	-0.8980	-0.5750
Glucose	-0.9164	0.9187	0.9254	1.0000	0.9224	-0.7095	-0.9079	-0.6423
Fructose	-0.9370	0.9715	0.7677	0.9224	1.0000	-0.5936	-0.8457	-0.7202
Methanol	0.6573	-0.6497	-0.7521	-0.7095	-0.5936	1.0000	0.4549	0.6247
Ethanol	0.9100	-0.8063	-0.8980	-0.9079	-0.8457	0.4549	1.0000	0.4979
Acetic acid	0.7358	-0.7380	-0.5750	-0.6423	-0.7202	0.6247	0.4979	1.0000
